# Editorial: Minimally invasive therapies and molecular mechanisms related to recurrence in hepatobiliary and gastric cancers

**DOI:** 10.3389/fonc.2023.1335049

**Published:** 2023-12-06

**Authors:** Manqiu Kang, Wei Chen, Yuming Jiang, Changchang Jia

**Affiliations:** ^1^ Department of Cell-Gene Therapy Translational Medicine Research Center, The Third Affiliated Hospital of Sun Yat-Sen University, Guangzhou, China; ^2^ Department of Hepatobiliary and Pancreatic Surgery, The First Affiliated Hospital, Zhejiang University School of Medicine, Hangzhou, China; ^3^ Department of Radiation Oncology, Wake Forest University School of Medicine, Winston-Salem, NC, United States

**Keywords:** gastric cancer, anastomotic leakage, hepatocellular carcinoma, primary hepatic neuroendocrine tumors, radiomics

This research aims to establish a platform for evaluating the clinical effectiveness and safety of minimally invasive therapies for liver, gallbladder, and gastrointestinal tumors. Additionally, it seeks to explore advanced non-invasive diagnostic methods for predicting tumor prognosis and unravel the molecular mechanisms associated with tumor recurrence in these areas. The study, enriched with cutting-edge research contributions, has successfully achieved its primary goals and formulated recommendations for advancing the treatment and prognosis of these tumors in the future.

For instance, He et al. conducted a retrospective analysis of clinical and pathological data in gastric cancer patients to investigate risk factors and outcomes of conservative treatment for anastomotic leakage (AL) following gastric resection. Their findings identified low serum albumin, diabetes, laparoscopic surgery, total gastrectomy, and proximal gastrectomy as predictive risk factors for anastomotic leakage. The study emphasizes a shift from surgical to combined conservative and endoscopic interventions in the treatment of anastomotic leakage, with technological advancements supporting the efficacy of endoscopic treatment. This study focuses on analyzing risk factors and treatment options for anastomotic leakage after gastric resection, offering crucial recommendations for managing postoperative complications that significantly impact postoperative survival and quality of life, particularly in the context of tumor diseases (1).

Furthermore, Liang et al. explored the relationship between lipid metabolism, the use of lipid-lowering medications, and liver cancer. Given the liver’s pivotal role in lipid metabolism and the rising prevalence of obesity (2, 3), understanding the connection between lipid metabolism, the utilization of lipid-lowering drugs, and the development of liver tumors becomes imperative. Employing Mendelian randomization analysis, the research establishes a causal relationship between individual serum lipid markers and the incidence of liver cancer. The study also employs drug target Mendelian randomization (DMR) analysis to assess the causal impact of LDL-c-lowering medications on the risk of liver cancer, suggesting that such drugs can mitigate the risk by reducing LDL-c levels. This study provides unique insights into predicting the occurrence of liver cancer and offers valuable recommendations for prevention.

Additionally, Chen et al. conducted a significant study using dual-region CT radiomics to predict FOXM1 expression and prognosis in hepatocellular carcinoma (HCC). Integrating FOXM1 expression with radiomics offers a fresh perspective on non-invasive tumor characterization, implying its potential in providing molecular insights. Within this Research Topic, a case report by Tang et al. details two cases of primary hepatic neuroendocrine tumors (PHNET). The report aims to document the characteristics of this rare liver tumor, advance comprehension of the disease, enhance diagnostic precision, and explore standardized diagnostic and treatment approaches.

Collectively, the articles within this Research Topic effectively align with its objectives and exhibit significant innovation. They emphasize the adoption of minimally invasive or non-invasive methods for the treatment and prognostication of liver, gallbladder, and gastrointestinal tumors, offering increased convenience in clinical diagnosis and treatment while mitigating patient discomfort. Pioneering reports, such as the use of Mendelian randomization analysis to establish the causal link between lipid-lowering drugs, LDL-c levels, and the development of liver cancer (Liang et al.), serve as exemplary models for future research endeavors. The study on radiomics, a fusion of imaging and molecular detection, represents a burgeoning research domain with promising applications in the management of advanced tumors (Chen et al.). This research has the potential to provide optimal solutions for addressing the clinical challenges encountered by patients with advanced tumors who cannot undergo invasive testing due to their physical condition, thereby offering a viable remedy for this clinical predicament (4).

## Author contributions

MK: Investigation, Writing – original draft. WC: Supervision, Validation, Writing – review & editing. YJ: Supervision, Writing – review & editing. CJ: Conceptualization, Supervision, Validation, Writing – original draft, Writing – review & editing.

## References

[1] Kamarajah SK Navidi M Griffin SM Phillips AW. Impact of anastomotic leak on long-term survival in patients undergoing gastrectomy for gastric cancer. Br J Surg (2020) 107(12):1648–58. doi: 10.1002/bjs.11749 32533715

[2] Ng M Fleming T Robinson M Thomson B Graetz N Margono C. Global, regional, and national prevalence of overweight and obesity in children and adults during 1980-2013: a systematic analysis for the Global Burden of Disease Study 2013. Lancet (2014) 384(9945):766–81. doi: 10.1016/S0140-6736(14)60460-8 PMC4624264 24880830

[3] Ten HM Pater L Storm G Weiskirchen S Weiskirchen R Lammers T. The hepatic lipidome: From basic science to clinical translation. Adv Drug Deliv Rev (2020) 159:180–97. doi: 10.1016/j.addr.2020.06.027 32615143

[4] Liu J Fang L Qi S Song Y Han L. Occult extracranial Malignancy after complete remission of pineal mixed germ cell tumors: a rare case report and literature review. BMC Pediatr (2023) 23(1):447. doi: 10.1186/s12887-023-04213-9 37679697 PMC10483866

